# Bismuth nitrate-induced microwave-assisted expeditious synthesis of vanillin from curcumin

**DOI:** 10.1186/2191-2858-2-15

**Published:** 2012-04-20

**Authors:** Debasish Bandyopadhyay, Bimal K Banik

**Affiliations:** 1 Department of Chemistry, The University of Texas-Pan American, 1201 West University Drive, Edinburg, TX 78539, USA

**Keywords:** Curcumin, Vanillin, Microwave, Bismuth nitrate, Fragrance

## Abstract

**Background:**

Curcumin and vanillin are the two useful compounds in food and medicine. Bismuth nitrate pentahydrate is an economical and ecofriendly reagent.

**Method:**

Bismuth nitrate pentahydrate impregnated montmorillonite KSF clay and curcumin were subjected to microwave irradiation.

**Results:**

Microwave-induced bismuth nitrate-promoted synthesis of vanillin from curcumin has been accomplished in good yield under solvent-free condition. Twenty-five different reaction conditions have been studied to optimize the process.

**Conclusion:**

The present procedure for the synthesis of vanillin may find useful application in the area of industrial process development.

## Background

**Figure 1:**
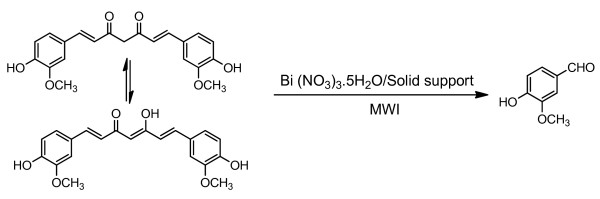
**Bismuth nitrate pentahydrate-induced simple synthesis of vanillin from curcumin under microwave irradiation**.

Curcumin, a polyphenol derived from *Curcuma longa *(commonly known as turmeric) is an ancient spice and therapeutic used in India for centuries to induce color in food and to treat a wide array of diseases. It has been demonstrated that curcumin has many beneficial pharmacological effects, including anti-inflammatory [1], antioxidant [2], antiviral [3], antiangiogenic [4] effects. Most importantly, curcumin possesses immense antitumorigenic effect. It prevents tumor formation in a number of animal models, including models of skin, colon, liver, esophageal, stomach, and breast cancer [5-8]. Curcumin has also demonstrated the ability to improve patient outcomes in Phase I clinical trials [9]. The potential application of curcumin as a chemopreventive agent in both animal and human studies has been demonstrated [10]. Very recently, curcumin has been reported [11] as a protectant against neurodegenerative diseases through chelation with iron. On the other hand, vanillin (4-hydroxy-3-methoxybenzaldehyde) is an important guaiacol derivative which is extremely selective inhibitor of aldehyde oxidase. It has been found that it acts as a substrate of this enzyme, and is metabolized by aldehyde dehydrogenase [12]. Because of the exceptionally widespread utilization of vanillin in the food, cosmetic, pharmaceutical, nutraceutical and fine chemical industries makes this compound as one of the most important aromas. As a result of these crucial properties, considerable attention has been devoted to the improvement of the production processes of vanillin [13]. We report herein an easy and extremely rapid one-step method for the preparation of vanillin from naturally occurring curcumin in the presence of bismuth nitrate under microwave irradiation (Figure 1).

## Methods

FT-IR spectra were registered on a Bruker IFS 55 Equinox FTIR spectrophotometer as KBr discs. ^1 ^H NMR (300 MHz) and ^13 ^C NMR (75 MHz) spectra were obtained at room temperature with JEOL-300 equipment using d_6_-DMSO as solvent. Analytical grade chemicals (Sigma-Aldrich Corporation, Milwaukee, USA) were used throughout the project. Deionized water was used for the preparation of all aqueous solutions.

## Results and discussion

In continuation of our research on environmentally benign reactions, we have been working on methodology development using microwave irradiation for many years. Using microwave irradiation technique, we have successfully developed several new organic methodologies which include stereoselective synthesis of β-lactams [14-16], synthesis of pyrroles [17-20], aza-Michael addition [21], and synthesis of quinoxalines [22]. On the other hand, we have demonstrated the catalytic activity of trivalent bismuth nitrate pentahydrate in a number of occasions. These experiments resulted in various methods that include nitration of aromatic systems [23-25], Michael reaction [26], protection of carbonyl compounds [27], deprotection of oximes and hydrazones [28], Paal-Knorr synthesis of pyrroles [29], hydrolysis of amide [30], electrophilic substitution of indoles [31,32], synthesis of α-aminophosphonates [33], and Biginelli condensation [34]. Our success in the bismuth nitrate-induced reaction has confirmed that this reagent acts as a Lewis acid. Bismuth nitrate pentahydrate is proved to be an effective reagent for the preparation of vanillin. However, Zn(NO_3_)_2_, Ca(NO_3_)_2_, LaNO_3_, NaNO_3_, ceric ammonium nitrate, and Cu(NO_3_)_2 _were also studied but without any success. Dry conditions and solvent-free methods along with commercial solvents without any purification were investigated in order to identify the best conditions for this reaction (Table 1). Reactions were performed at high temperature using Dean-Stark water separator, traditional reflux, and conventional kitchen microwave-induced methods. Solid surfaces such as florisil, silica gel, molecular sieves, montmorillonite KSF clay, and neutral alumina were used as solid support in the reaction. It has been found that montmorillonite KSF clay is the best solid surface (entries 4, 9, and 19) among all others. 

**Table 1:** Bismuth nitrate pentahydrate-induced simple synthesis of vanillin from curcumin following Figure 1

Entry	Solid surface	Method/solvent	Yield (%)
1	Florisil	Dean-Stark/Benzene	NR^a^
2	Silica gel	Dean-Stark/Benzene	NR
3	Molecular sieves	Dean-Stark/Benzene	NR
4	KSF clay	Dean-Stark/Benzene	10
5	Neutral alumina	Dean-Stark/Benzene	NR
6	Florisil	Reflux/DCM	NR
7	Silica gel	Reflux/DCM	NR
8	Molecular sieves	Reflux/DCM	NR
9	KSF clay	Reflux/DCM	34
10	Neutral alumina	Reflux/DCM	15
11	Florisil	Dry^b^	NR
12	Silica gel	Dry	NR
13	Molecular sieves	Dry	NR
14	KSF clay	Dry	NR
15	Neutral alumina	Dry	NR
16	Florisil	Microwave/solvent free	60
17	Silica gel	Microwave/solvent free	54
18	Molecular sieves	Microwave/solvent free	45
19	KSF clay	Microwave/solvent free	77
20	Neutral alumina	Microwave/solvent free	61
21	Florisil	Reflux/Benzene	NR
22	Silica gel	Reflux/Benzene	NR
23	Molecular sieves	Reflux/Benzene	NR
24	KSF clay	Reflux/Benzene	NR
25	Neutral alumina	Reflux/Benzene	NR

^a^No reaction

^b^Without microwave irradiation, room temperature

## Experimental

Curcumin (1 mmol), bismuth nitrate pentahydrate (0.75 equivalent), and solid support (500 mg) were mixed in dichloromethane (4 mL) and the solvent was evaporated by rotavapor. The mixture was irradiated in kitchen microwave and the reaction was monitored by TLC. After completion of the reaction (Table 1), the reaction mixture was extracted with dichloromethane and basified with saturated aqueous sodium bicarbonate solution. The organic layer was then washed with brine and water successively, dried with anhydrous sodium sulfate. The pure product (77%) was isolated by flash chromatography over silica gel.

### 4-hydroxy-3-methoxybenzaldehyde (vanillin)

Light yellow crystals; Mp: 82-83°C, IR (KBr disk, cm^-1^): 3176, 1679, 1597, 1512, 1426, 1385, 1112, 814, 710; ^1 ^H NMR (d_6_-DMSO, 300 MHz) δ: 9.86 (s, 1 H), 8.09 (m, 2 H), 7.57 (s, 1 H), 3.96 (s, 1 H). ^13 ^C NMR (d_6_-DMSO, 75 MHz) δ: 190.98, 151.33, 148.08, 137.57, 128.28, 121.47, 113.05, and 57.34.

## Conclusions

In summary, a new and simple method for the synthesis of vanillin from naturally occurring curcumin has successfully been investigated. Trivalent bismuth nitrate-induced synthesis of vanillin has successfully been carried out under various conditions and the formation of a single product (4-hydroxy-3-methoxybenzaldehyde) has been observed in variable yields. The exploratory results described herein confirm that bismuth nitrate pentahydrate is the reagent of choice for the oxidative cleavage of curcumin to vanillin in the absence of any solvent under microwave-irradiation condition (entry 19). Importantly, no aromatic nitration and rearrangement of curcumin or vanillin has been observed with bismuth nitrate. A selective oxidation of the alkene bond of curcumin to vanillin has taken place. Considering the structure of vanillin and the conditions of the experiments, one can expect further oxidation of the aromatic aldehyde group or nitration of the aromatic system might be other possibilities. However, it is interesting to note that such reactions although feasible, but vanillin is the only isolated product. On the basis of these important and selective observations, this method will find very useful applications in industrial chemistry.

## Competing interests

The authors declare that they have no competing interests.

## Authors' contributions

DB performed the reactions and structure elucidation of the product. All authors read and approved the final manuscript.
